# W. W. Allport, M.D., D.D.S.

**Published:** 1893-05

**Authors:** 


### Obituary.

### W. W. ALLPORT, M.D., D.D.S.

Dr. W. W. Allport died at Chicago on Tuesday, March 21, 1893. His death was due to meningitis preceded by erysipelas.

The death of this remarkable man has increased rather than diminished the interest felt in his personality during his life. He was one of the very few connecting links binding the new order of dentistry with the transition period. His long life of activity and earnest advocacy of a higher standard of work made him a prominent figure in all the labor of the profession. His pronounced views and marked ability in giving them utterance invariably claimed attention. He was emphatically a leader among men. He began the work of his life at such an early period that but few in active practice at the present time can realize that Dr. Allport has been an authority for fully forty years. The writer of this can well recall the interest felt in any suggestions from his pen as early as 1854. From this time to his last sickness he has been untiring in this work.

The last time the writer met him was at the meeting of the American Dental Association, at Niagara Falls, August, 1892. He seemed then to be as vigorous and as full of life as any present. Few will forget his lucid explanation at that meeting of the use of soft gold, and the energy with which he entered into the collection of funds for his old friend and co-worker, Dr. Dwinelle.

He has passed away, and with his departure will go much of the antagonistic feeling engendered by his honest and vigorous protests against what he considered wrong, and in its place will come a clearer conception of his true character.

We have not space to enlarge more fully upon this theme, but leave him with the conviction that dentistry has never had a better exponent or one more fully fitted to build from its disorganized elements a profession worthy of the respect of men.

We are indebted to the *Chicago Tribune* for the following details of his life:

"Dr. Walter Webb Allport was born at Lorain, Jefferson County, New York, in June, 1824. He was of English descent, and Sir James Allport, one of the greatest railroad men in England, and Dean Allport were his cousins. In 1844 he entered the office of Professor Amasa Trowbridge, at Watertown, to study medicine. In 1846 he determined to devote his attention to dentistry, and in 1853 entered the New York Dental College in the double capacity of student and demonstrator. He graduated from that institution in 1853, removed to Chicago in 1854, and has practised his profession here ever since. He married Miss Sarah Maria Haddock at Watertown in 1847. Dr. Allport's beginning in Chicago was a modest one. A room seven by eight feet in size on Lake Street furnished ample accommodations for him and a physician. Dr. Allport occupied one corner of the room. His operating-case consisted of a board nailed across the angle and covered with a newspaper, and his chair he had rented from a barber. His earnings for the first two months were twenty dollars and thirty-nine dollars, respectively. A few years ago he discovered an old lady in one of whose teeth was the first filling that he made in this humble office, showing that from the first he had been a skilful operator.

"In 1858, Dr. Allport was elected President of the Western Dental Society; in 1860 he was elected the first Chairman of the American Dental Association; in 1865 he was elected President of the American Dental Convention; and in 1886 he was elected President of the American Dental Association. In 1881, Rush Medical College conferred on him the honorary degree of M.D., and for many years he was Emeritus Professor of Dental Surgery in that institution and in the Chicago Dental College. He was the means of creating the Dental Section in the Ninth International Medical Congress which met in Washington in 1887, and was made Vice-President of the Section. He was one of the organizers of the Chicago Microscopical Society, and for a long while its President. He was largely instrumental in the organization of the American

Dental Association and in projecting the World's Columbian Dental Congress to be held in Chicago this summer. He was also the editor for two years of the *People's Dental Journal.*

"Dr. Allport was a man of venerable and distinguished appearance, with a commanding figure, a noble head, and features expressive of intelligence, precision, honesty, and courage. Like all positive characters he had devoted friends and unforgiving enemies, but both alike respected his integrity and his independence. He was a member of Grace Episcopal Church, and was at one time an active Mason, but latterly he belonged to no club and no secret order. He always found his delight in his own home with his large and interesting family."

The following analysis of his character, furnished us by Dr. E. S. Talbot, will be read with interest. Dr. Talbot's long intimacy with Dr. Allport gives especial value to his views at the present time.

"Dr. Allport, in his way, was one of the most remarkable men in the profession. He detested fraud, underhand work, and wire-pulling for the sake of office. He was continually fighting these things, hence he gained for himself many enemies. He was not in the strict sense of the word a scientific man, although he did considerable original labor in the early days. He was always anxious to do his part to help along the cause. One of his methods was to try to organize societies on a strictly honest basis. His great work was in the American Dental Association. In the International Medical Congress he overthrew all schemes and made a grand success of the Dental Section.

"Dr. Allport did not care for notoriety, although many regard this as one of his faults. His character in this respect I am familiar with, as he frequently consulted me in regard to what position he should take in a given case. He did not care to become President of the Dental Congress, and often said if he could not get the position without politics, he would not have it. Six years ago he saw that there must be a Dental Congress in this country, although he was opposed to it, as he believed that the International Medical Congress was all that was necessary, yet he went to work with a view of making it a scientific affair, far above what the ordinary dentist had anticipated; but not being satisfied with the course pursued, he became discouraged and let the matter drop. Dr. Allport, to my knowledge for the past ten years, has spent his time to help the profession, and not for personal gain. He refused the presidency of the Dental Section of the Fourth International Medical Congress, because he was satisfied with what he had accomplished in the organization, and did not care for the honor of the position. He was a man far above the average dentist, and could see farther into the future, both in dentistry and business affairs, than any man I have ever seen. Our best men consulted him in regard to business and investments in real estate. He was a thorough gentleman in every respect. It was not generally known, but he possessed a kind, sympathetic nature. I have seen tears in his eyes many times in sympathy for the misfortunes of others.

"In 1874, Dr. Allport delivered the anniversary address at the American Academy of Dental Science, Boston, advocating the divorcement of prosthetic from operative dentistry. He foresaw years ago, that in large cities dentistry must be divided into specialties; that mechanical dentistry must become a special and a distinct branch, not requiring the same amount of study required of operative dentistry.

"Dr. Allport's best literary work was done in his earlier days before I knew him. He edited a journal called the *People's Dental Journal*, and at that date (1863–64) it was considered a valuable production in that particular line of literature. His articles were read by all dentists with great enthusiasm, since they contained so much that was of interest to practitioners. I am told by older members of the profession that the *Journal* was glanced over as soon as received to see if there was something from his pen. He was chairman of the Section of Dental and Oral Surgery twice, and had read the following able papers before that body, published in the *Journal of the American Medical Association:* December 8, 1883, page 637, 'A Case of Amaurosis dependent upon Dental Irritation;' February 7, 1885, page 147, 'A Case of Vicarious Catamenial Hemorrhage of the Gum, with Recession of the Gingival Margin and Alveolar Process, the Result of Amenorrhœa;' August 17, 1889, page 229, 'Facial Neuralgia consequent upon Pregnancy.' It will be seen by the subjects that the doctor was a very broad man in his study, and was well up in a general way upon all diseases of the body. His papers upon dental subjects will be found in the *Dental Cosmos* and the Transactions of the American Dental Association."

The closing services were held at his home Wednesday, March 22. The interment took place at Graceland Cemetery, Chicago.

